# The appearance of a single left atrial tachycardia as two different types on preoperative surface and intracardiac ECG of pacemaker due to progressive cardiac conduction disturbance

**DOI:** 10.1002/ccr3.2943

**Published:** 2020-07-15

**Authors:** Yusuke Sakamoto, Hiroyuki Osanai, Hiroto Uno, Hideki Kurokawa, Shun Kondo, Kotaro Tokuda, Takahiro Kanbara, Yoshihito Nakashima, Hiroshi Asano, Masayoshi Ajioka

**Affiliations:** ^1^ Department of Cardiology Tosei General Hospital Seto Japan

**Keywords:** ablation, atrial tachycardia, electrocardiography, interatrial conduction disturbance, progressive cardiac conduction disturbance

## Abstract

We treated a patient with PCCD whose single left AT appeared as two different types on preoperative surface and intracardiac ECG from a pacemaker. The diagnosis was hindered by the fact that the conduction block encompassed interatrial block and the pacemaker A‐wave was captured at the right atrial appendage.

## Graphical abstract

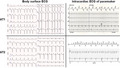

## 1 INTRODUCTION

We describe a patient with atrioventricular block who developed tachycardia after pacemaker implantation. Preoperative body surface and intracardiac electrocardiography (ECG) from a pacemaker two types of tachycardia; however, only left atrial tachycardia was present. Conduction disturbance between left and right atria gave the appearance of two different types.

Progressive cardiac conduction disturbance (PCCD) has a strong genetic component, and in addition to progressive abnormalities of the excitation‐conduction system, patients may also exhibit supraventricular and ventricular arrhythmias, leading to impaired cardiac function.^1^, ^2^

Here, we present the case of a patient with PCCD who also suffered from atrial tachycardia before ablation was performed. Her condition was difficult to diagnose with surface electrocardiography (ECG) and intracardiac ECG from a pacemaker.

## 2 CASE HISTORY

A 54‐year‐old woman was referred to our hospital after asymptomatic complete atrioventricular block was identified during a health checkup in 2017. At the time of referral, she also had sick sinus syndrome with serious sinus bradycardia. She had a family history of pacemaker implantation, as her grandmother, mother, and maternal aunt had all been fitted with pacemakers, and her brother was undergoing investigations at another hospital to identify the cause of fainting spells.

An echocardiogram prior to pacemaker implantation showed good cardiac contraction. Organic heart disease, skeletal myopathy, sarcoidosis, autoimmune disease, atherosclerotic disease, and other conditions were ruled out by cardiac magnetic resonance imaging and other investigations. PCCD was diagnosed, and the patient was fitted with a pacemaker.

**Figure 1:**
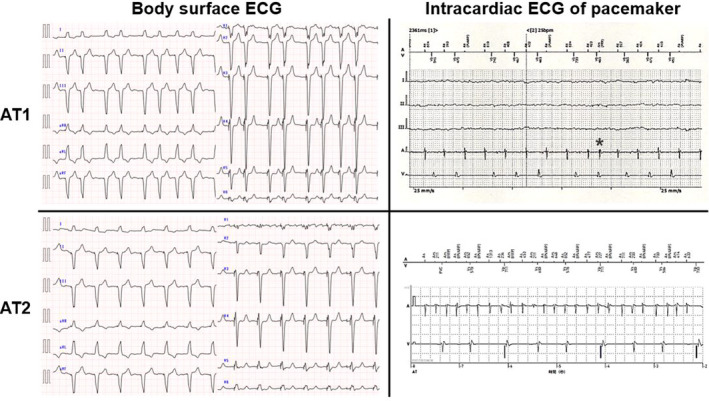
Surface ECG and intracardiac ECG recorded on the pacemaker showing AT1 and AT2. The asterisk (*) shows where an A‐wave with different timing was recorded

About 2 years after pacemaker implantation, the patient developed atrial tachycardia (AT). Surface ECG and intracardiac ECG from the pacemaker showed two types of atrial excitation. Although both types of AT had a similar P‐wave morphology, the P‐wave rate was approximately 150 bpm on the V1 lead in AT1 on the surface ECG, with a tachycardia cycle length (TCL) of approximately 400 ms on the intracardiac ECG from the pacemaker. An A‐wave with different timing was also recorded on the intracardiac ECG from the pacemaker during AT1. In comparison, in AT2 on surface ECG, the P‐wave rate was approximately 300 bpm on the V1 lead, with a TCL of approximately 200 ms on the intracardiac ECG from the pacemaker (Figure 1).

**Figure 2:**
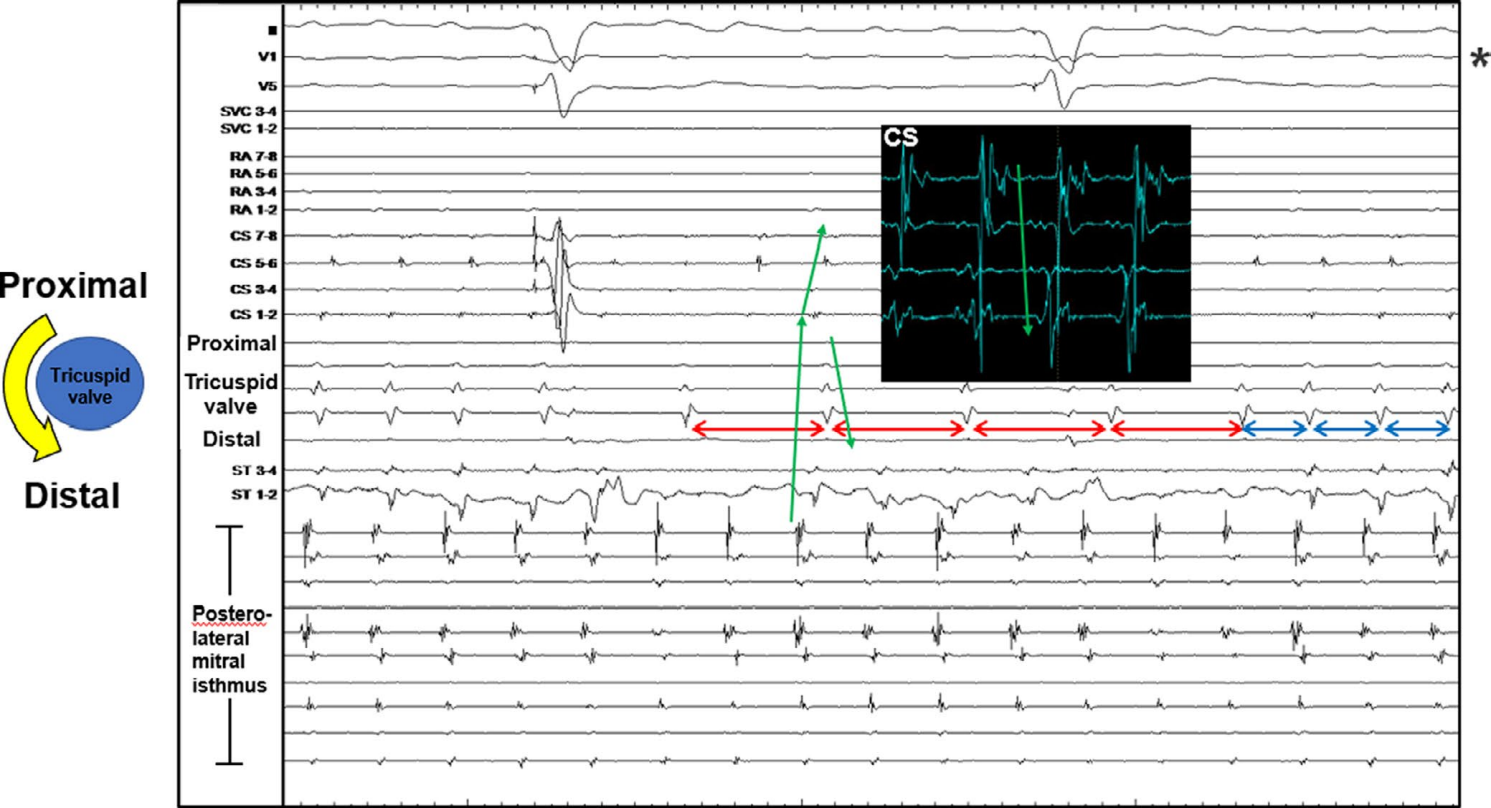
Intracardiac ECG during tachycardia. The electrode catheter placed in the tricuspid valve was positioned as shown in the diagram. The electrode catheter placed in the left atrium was positioned in the posterolateral mitral isthmus. At the V1 lead indicated by the asterisk (*), 2:1 block associated with conduction disturbance from the left atrium to the right atrium was reflected in the surface ECG

The frequency of tachycardia episodes gradually increased; therefore, catheter ablation was performed on March 2019. The patient had not taken any antiarrhythmic drugs until the time of the ablation. The patient was started on an oral anticoagulant (DOAC; apixaban 10 mg) prior to ablation, and left atrial thrombus was excluded by contrast‐enhanced CT and measurement of coagulation markers using blood tests. At the start of ablation, intracardiac ECG showed continuous AT with a TCL of 200 ms Figure 2 shows intracardiac ECG during an episode of tachycardia. This showed that during tachycardia, conduction from the left atrium to the right atrium intermittently exhibited 2:1 conduction block. This was also reflected in the surface ECG. Left AT was propagated proximal to distal on the endocardial side of the mitral annulus and from distal to proximal in the coronary sinus musculature. There was a conduction disturbance observed between these areas. Excitation from the left atrium to the right atrium exhibited retrograde conduction via Bachmann's bundle alone and was propagated from the top to the bottom of the right atrium. Retrograde conduction via Bachmann's bundle was associated with tachycardia, and a 2:1 block was apparent. Although two types of tachycardia had been inferred from preoperative surface ECG and intracardiac ECG from the pacemaker, only a single type of left AT was present. It had appeared as two different types due to the conduction disturbance between the right and left atria. The A‐wave with different timing observed on the preoperative intracardiac ECG from the pacemaker during AT1 was considered to have propagated only a single beat from the left atrium to the right atrium at 1:1.

**Figure 3:**
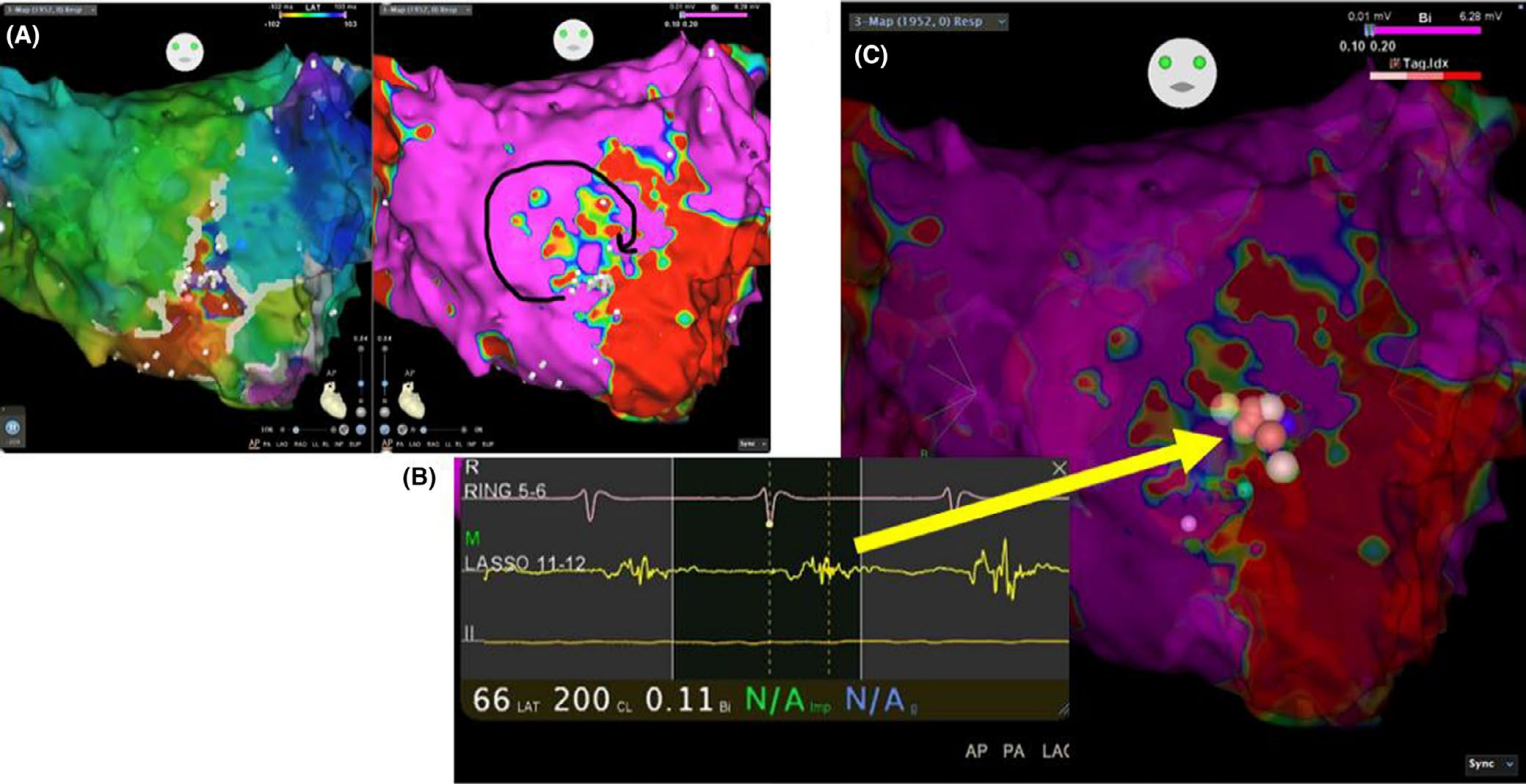
3D map generated by CARTO3. A, Ripple map generated by CARTO3 shows reentry rotating clockwise around the left atrial anterior wall. The channel for the tachycardia was believed to run between a scar on the anterior wall and the mitral annulus. B, Fragment potential identified at the site believed to constitute the channel. C:The passage of a current through this site terminated tachycardia, which could no longer be induced following further ablation of the surrounding area

Voltage mapping of the left atrium using CARTO3 revealed a low‐voltage zone (LVZ) in the anterior wall only. Reentry that rotated clockwise around the left atrial anterior wall was identified on a ripple map. The channel for the tachycardia was believed to run between a scar on the anterior wall and the mitral annulus. There was fragment potential between the LVZ in the anterior wall and the mitral annulus, and the passage of a current through this site terminated tachycardia, which was no longer induced following further ablation (Figure 3). There has been no post‐treatment recurrence of AT.

## 3 DISCUSSION

According to a 2013 joint statement by the Heart Rhythm Society, European Heart Rhythm Association, and the Asia‐Pacific Heart Rhythm Society, PCCD is diagnosed in the presence of unexplained progressive conduction abnormalities in young (<50 years) individuals with structurally normal hearts in the absence of skeletal myopathies, especially if there is a family history of PCCD.^3^ Although there are no established diagnostic criteria in Japan, the Japan Circulatory Society's Guidelines for Diagnosis and Management of Inherited Arrhythmias (JCS 2017) suggest that the following four conditions should be met as diagnostic criteria for PCCD^4^: (a) ECG findings indicative of progressive conduction disorder (bifascicular block or Mobitz type II or worse atrioventricular block); (b) Syncope or other bradycardia symptoms, or a previous history of or family history of pacemaker implantation; (c) <70 years old at diagnosis (d); Exclusions at diagnosis: organic heart disease, skeletal myopathy, sarcoidosis, autoimmune disease, or atherosclerotic disease. Patients with cardiomyopathy or skeletal myopathy are not excluded if these were predated by the symptoms or pathophysiology of bradycardia or if the involvement of bradycardia is suspected. Our patient was diagnosed with PCCD because she was <70 years old, suffered from sick sinus disease in addition to atrioventricular conduction disorder despite the absence of underlying heart disease, and had a strong family history of pacemaker implantation. Although ablation successfully treated the AT, electrophysiological studies revealed an interatrial conduction disturbance and conduction disturbance between the pericardial side of the atrium and the coronary sinus musculature. Therefore, the possibility of a future electrical isolation of the left atrium is a matter of concern.

The AT that occurred in this patient was macroreentrant tachycardia with a scar in the left atrial anterior wall as its channel. A previous study described patients without previous atrial surgery or catheter ablation as cases of scar‐related left atrial anterior wall reentry.^5^ In that study, although the scars in the left atrial anterior wall had various causes, most patients were elderly. In our patient, computed tomography confirmed that the position of the aortic cusp coincided with the position of the left atrial anterior wall scar, suggesting that the scar might have formed due to its pressure, but it is conceivable that scar formation in young patients might also be the result of PCCD.

## 4 CONCLUSION

We treated a patient with interatrial conduction block due to PCCD in whom a single left AT appeared as two different types on preoperative surface ECG and intracardiac ECG from a pacemaker. The diagnosis was hindered by the fact that the conduction block also encompassed interatrial block and the pacemaker A‐wave was captured at the right atrial appendage.

## CONFLICT OF INTEREST

This report was not funded by any agencies in the public, commercial, or not‐for‐profit sectors.

## AUTHOR CONTRIBUTIONS

YS: conception and design of study, acquisition of data, analysis and interpretation of data, drafting the manuscript, approval of the version of the manuscript to be published. HO: conception and design of study, approval of the version of the manuscript to be published. HU: technical help, writing, and editing assistance. HK: technical help, writing, and editing assistance. SK: technical help, writing, and editing assistance. KT: technical help, writing, and editing assistance. TK: technical help, writing, and editing assistance. YN: technical help, writing, and editing assistance. HA: technical help, writing, and editing assistance.
